# Advancing Engineered Plant Living Materials through Tobacco BY-2 Cell Growth and Transfection within Tailored Granular Hydrogel Scaffolds

**DOI:** 10.1021/acscentsci.4c00338

**Published:** 2024-05-01

**Authors:** Yujie Wang, Zhengao Di, Minglang Qin, Shenming Qu, Wenbo Zhong, Lingfeng Yuan, Jing Zhang, Julian M. Hibberd, Ziyi Yu

**Affiliations:** † State Key Laboratory of Materials-Oriented Chemical Engineering, College of Chemical Engineering, Nanjing Tech University, 30 Puzhu South Road, Nanjing 211816, People’s Republic of China; ‡ Department of Plant Sciences, University of Cambridge, Downing Street, Cambridge CB2 3EA, U.K.; § Earlham Institute, Norwich Research Park, Norwich NR4 7UG, U.K.

## Abstract

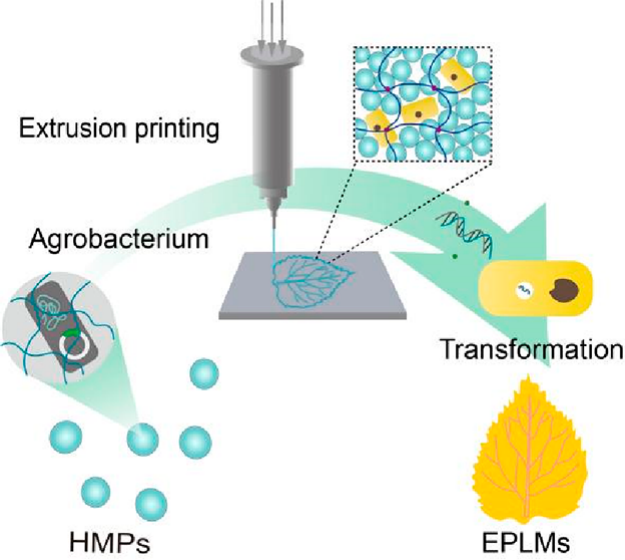

In this study, an innovative approach is presented in the field of engineered plant living materials (EPLMs), leveraging a sophisticated interplay between synthetic biology and engineering. We detail a 3D bioprinting technique for the precise spatial patterning and genetic transformation of the tobacco BY-2 cell line within custom-engineered granular hydrogel scaffolds. Our methodology involves the integration of biocompatible hydrogel microparticles (HMPs) primed for 3D bioprinting with *Agrobacterium tumefaciens* capable of plant cell transfection, serving as the backbone for the simultaneous growth and transformation of tobacco BY-2 cells. This system facilitates the concurrent growth and genetic modification of tobacco BY-2 cells within our specially designed scaffolds. These scaffolds enable the cells to develop into predefined patterns while remaining conducive to the uptake of exogenous DNA. We showcase the versatility of this technology by fabricating EPLMs with unique structural and functional properties, exemplified by EPLMs exhibiting distinct pigmentation patterns. These patterns are achieved through the integration of the betalain biosynthetic pathway into tobacco BY-2 cells. Overall, our study represents a groundbreaking shift in the convergence of materials science and plant synthetic biology, offering promising avenues for the evolution of sustainable, adaptive, and responsive living material systems.

## Abstract 2

Utilizing *Nicotiana tabacum* BY-2 cells within custom hydrogel scaffolds, this research demonstrates the creation of genetically modified engineered plant living materials with specific geometries and functionalities. Innovative granular hydrogel microparticles facilitate cell growth and DNA transformation, leading to those materials with unique pigmentation and fluorescent patterns. This advancement opens new avenues in bioengineering, offering diverse applications in bioengineering and material science.

## 1 Introduction

Engineered living materials represent an innovative intersection of biology and engineering, heralding a new area of materials science and offering an exciting frontier in technological advancement.^1^ These materials integrate living cells with nonliving matrices to create materials with tailored functions, carefully designed to harness the unique capabilities of biological systems.^2^ Unlike conventional materials, engineered living materials can grow, self-repair, adapt to environmental changes, and exhibit responsive behaviors.^3−5^ By merging the traits of living organisms with the stability and durability of nonliving substances, engineered living materials offer unprecedented potential for a range of applications, from sustainable construction and environmental remediation to advanced medical therapies and progressive biomanufacturing.^6,7^ In the pioneering phases of engineered living materials, the living organisms were primarily derived from bacterial and fungal cells, known for their rapid growth and biofilm formation abilities.^8−13^ Utilizing synthetic biology to modify microorganisms, combined with the use of 3D printing to shape living inks into intricate structures, has opened up new and promising paths in the developing field of engineered living materials.^14^

Recent advancements in plant suspension cultures have ignited enthusiasm for their use in creating engineered living materials, given their rapid growth and potential to construct plant-based cell factories.^15−18^ Their inherent structural rigidity from cellulose-rich cell walls, coupled with the ability to perform photosynthesis, sets the stage for an autonomous and energy-efficient system.^19^ Additionally, the unique secondary metabolisms and the ability to be genetically manipulated render plants to be an ideal platform for producing a myriad of beneficial secondary metabolites and pharmaceutical proteins.^20,21^ These advantages, combined with their responsiveness to environmental stimuli and biodegradability, position plant-based engineered living material as a compelling option in expanding the landscape of bioengineered materials. Pioneering efforts in this field have led to the creation of self-mending living hydrogels, which integrated chloroplasts as carbon-fixing photocatalysts.^22^ The outcomes of photosynthesis in these setups can alter the microstructures of 3D-printed living materials. Additionally, there has been advancement in producing tissue-like living materials through the hydrogel-based growth of plant cells.^23^ However, the examples described above cannot reach the level of genetically programmed plant cells, resulting in a deficiency of engineered complex behaviors within those living materials. The intricate shapes and structures are essential for the efficacy and function of plant-based engineered living materials. Therefore, fabricating engineered living materials from plant cells that manage microstructure and anisotropy in three dimensions is still a relatively unexplored domain.

In this work, we present a class of engineered plant living materials (EPLMs) that can be made into required geometries and functionalities via the growth and transfection of plant cells within tailored granular hydrogel scaffolds. The *Nicotiana tabacum* Bright Yellow-2 (BY-2) cell line was used as a model system in this study to demonstrate the construction of cell-laden hydrogels into EPLMs using 3D bioprinting technologies. To ensure plant cell viability and bioprinting, we designed and prepared biocompatible granular hydrogel microparticles (HMPs) with well-defined rheological properties. HMPs represent an innovative frontier in the realm of 3D printing, leveraging the physical interconnection of jammed microparticle dispersions to produce a distinctive stress-yield flow characteristic. The jammed HMPs and a hydrogel precursor, gelatin methacrylate (Gel-MA), are then mixed with tobacco BY-2 cells to create bioinks, allowing cells’ growth and division to be studied within a customizable hydrogel microenvironment. The tailored granular hydrogel scaffolds not only act as supporting structure for the growth of living materials but also, when loaded with *Agrobacterium*, function as vehicles to introduce foreign DNA into tobacco BY-2 cells. *Agrobacterium*-loaded HMPs (Agro-HMPs) enable in situ transformation of tobacco BY-2 cells within the same hydrogel scaffold, where subsequent cell growth and secondary metabolite productions can occur. EPLMs producing a class of plant pigments called betalains have been generated to demonstrate the ability and potential of constructing genetically modified EPLMs with bespoken structures and functions. The generated EPLMs display distinct pigmentation and fluorescent patterns through spatially controlled 3D printing of tobacco BY-2 cells and Agro-HMPs, showcasing the versatility of constructing multifunctional EPLMs using the current platform.

## 2 Results and Discussion

**Figure 1:**
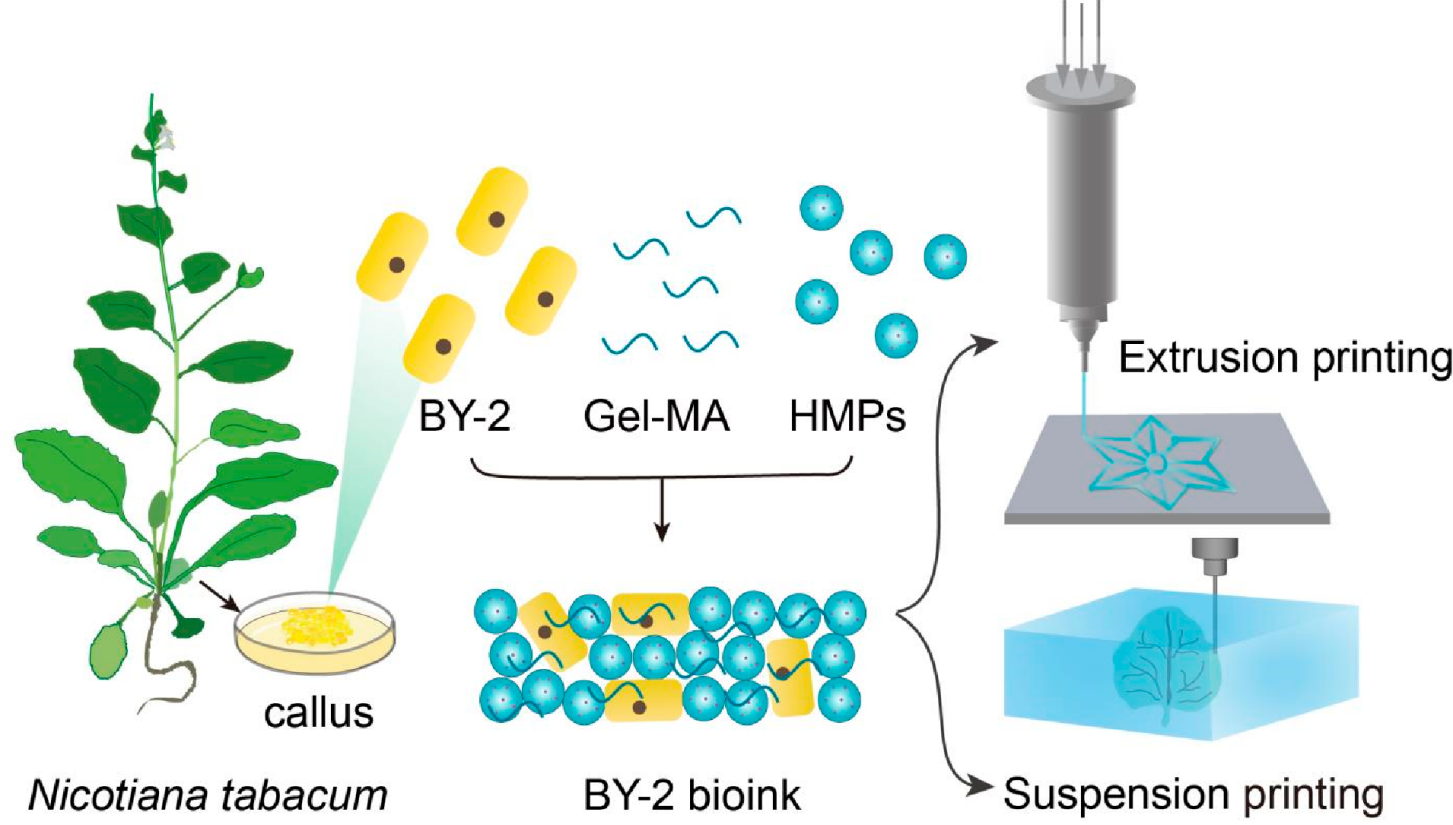
Schematic diagram of the 3D bioprinting of PLMs. BY-2 cell line derived from *Nicotiana tabacum*, HMPs, and Gel-MA were mixed in appropriate ratios to form BY-2-loaded bioinks, which can be printed into 3D structures via extrusion or suspension printing.

We started by designing granular hydrogel scaffolds for the bioprinting of plant living materials (PLMs). The BY-2 bioink that was used to print PLMs via direct-ink-writing consisted of three components: (i) HMPs in a close-packed condition as a discrete phase, forming the foundation of the bioink; (ii) tobacco BY-2 cells, which are one of the most widely used plant suspension cell lines derived from *N. tabacum* and can be maintained in liquid cultures; and (iii) hydrogel precursors Gel-MA infiltrated into the void space as a continuous phase, forming a polymer network between HMPs and BY-2 cells (Figure 1). Owing to their shear-thinning and self-healing properties, jammed HMPs and hydrogel precursors are uniquely suited for bioink applications, allowing stable filament extrusion in both extrusion printing and suspension bioprinting.

**Figure 2:**
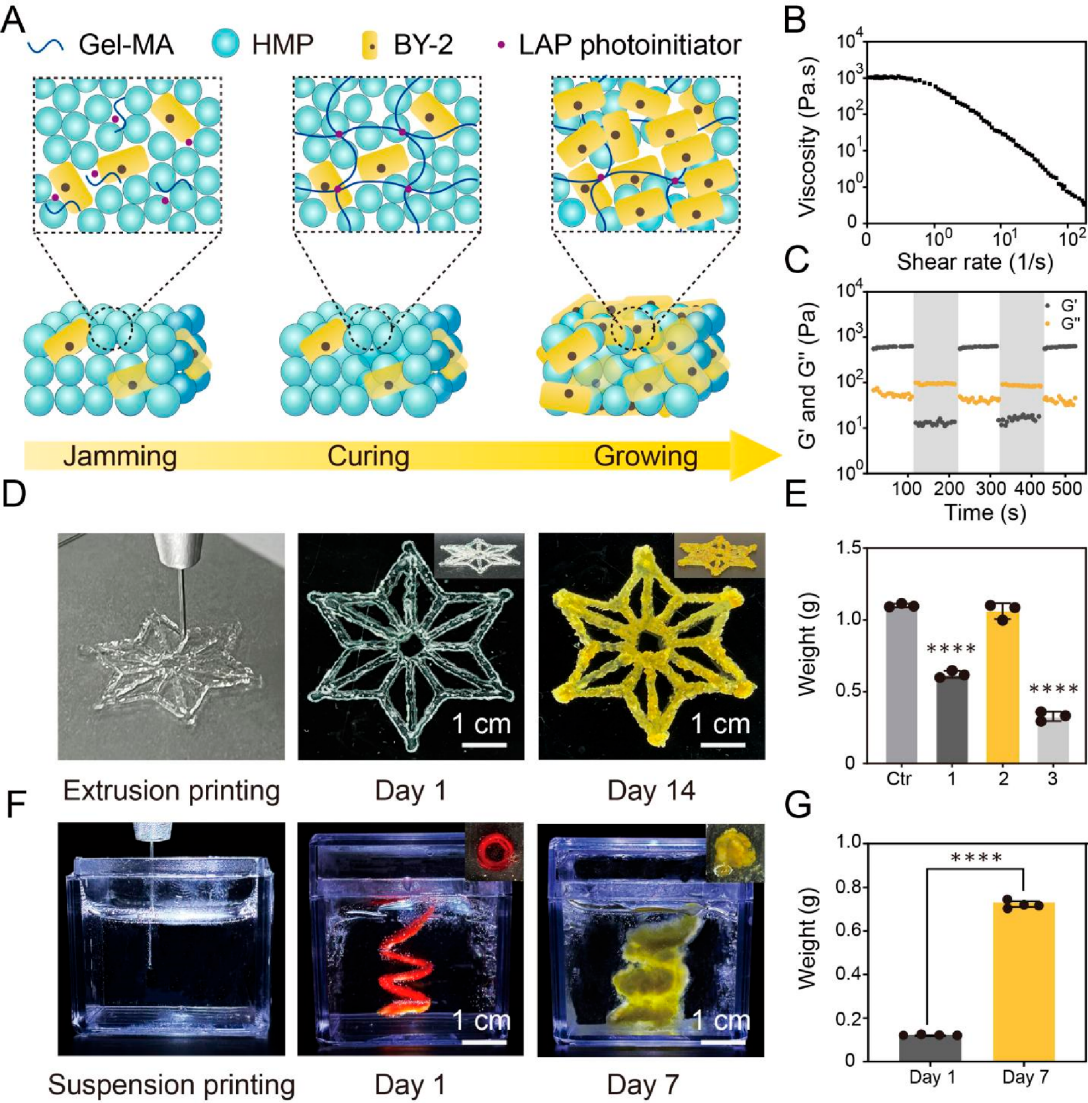
Characterization of the printing properties of BY-2 bioinks and subsequent cell growth. (A) Generation of PLMs involves the stacking of Gel-MA, HMP, and BY-2 cells into printable bioinks, curing of printed bioinks into rigid scaffolds via photo-cross-linking, and growth of BY-2 cells within scaffolds. (B) Apparent viscosity of jammed BY-2 bioink as a function of shear rate. (C) Self-healing properties through low (unshaded, 1% strain, 1 Hz) and high (shaded, 1000% strain, 1 Hz) strain cycles. (D) Extrusion printing of a snowflake-like PLM and its growth in 14 days. (E) Weight increases of extrusion-printed PLMs after 14 days with different ratios of HMP supplementation. A mixture of BY-2 and Gel-MA solution was first made by mixing the same amount of BY-2 cells with 500 μL of Gel-MA (10 wt %). The mixture was then mixed with different volumes (v/v) of HMPs as shown on the *x* axis. Ctr, BY-2 growing on MS solid media. Significance was calculated by one-way ANOVA, and **** indicates *p***<** 0.0001. (F) Suspension printing of a spiral-like PLM and its growth in 7 days. (G) Suspension-printed PLM weights at day 1 and day 7 after printing. Significance was calculated by single sample *t* test, and **** indicates *p***<** 0.0001.

Figure 2A illustrates the fabrication process of PLMs, which includes the jamming and curing of HMPs, followed by the cultivation of BY-2 cells within the granular hydrogel scaffolds. Gel-MA HMPs were first generated using a microfluidic device with a photo-cross-linking process. The generated HMPs were uniform in size, with diameters consistently ranging from 110 to 140 μm (Figure S2). Scanning electron microscopy (SEM) images of HMPs revealed their porous structure (Figure S3). Subsequently, generated HMPs were transferred from oil into a Gel-MA solution and then mixed with the BY-2 cells. The mixture was then subjected to “jamming” by removing the aqueous media from the particles and cells, creating an extrudable ink with microgel components and BY-2 cells distinctly visible under a microscope (Figure S4).

BY-2 bioinks were characterized rheologically to assess their printability for direct-ink-writing. The bioinks exhibited a shear-thinning behavior, wherein the viscosity decreased with increasing shear rate (Figure 2B). The bioinks demonstrated elastic hydrogel properties at low strains but yielded higher strains (Figure S5). Additionally, BY-2 bioinks underwent a swift and reversible transition from a solid-like elastic state to a liquid-like viscous state when subjected to high strain in oscillatory strain sweeps (Figure 2C). This behavior, probably caused by the disruption of contacts between HMPs at higher strains, showcased the capability of BY-2 bioinks to flow effectively during extrusion and swiftly stabilize following deposition, mirroring the typical properties of microgel suspensions. In addition, photorheology with blue light curing (405 nm) showed that the *G*′ increased from 4.37 ± 0.35 to 546.83 ± 131.33 Pa (Figure S5), demonstrating that the bioinks could be transformed from physical gels into a chemically cross-linked scaffold under light irradiation.

A three-axis extrusion printer, equipped with a conical precision nozzle (770 μm inner diameter), was utilized to demonstrate two bioprinting methods of PLMs, including a layer-by-layer bioprinting on a surface and a suspension technique within a supporting bath. In the layer-by-layer bioprinting method, a filament with a diameter about 750 μm was printed onto a cryogenic surface, creating a “snowflake” pattern, and then underwent photo-cross-linking to enhance structural stability (Figure 2D). To optimize BY-2 and HMPs ratios for PLM growth, a mixture of BY-2 and Gel-MA solution was first made by mixing 0.22 ± 0.01 g of BY-2 cells sampled from solid culture medium with 500 μL of Gel-MA (10 wt %); then the mixture was mixed with different volumes (v/v) of HMPs (Figure 2E). The highest biomass increase was obtained by supplementing HMPs into BY-2 and Gel-MA solution in a 2:1 ratio, namely, from 1.19 ± 0.07 g (day 1) to 2.25 ± 0.05 g (day 14). As the PLMs were incubated, BY-2 cells proliferated within the granular hydrogel scaffold, leading to the PLMs becoming visibly denser, as evidenced by a yellow coloration (Figure 2D, Figures S6, S8). Additionally, the growth of the plant cells was homogeneous throughout the material (Figure S10). The printed PLMs consistently retained their structural integrity and mechanical rigidity throughout the growth period (Figure S7). Other geometrically complex architectures could also be printed through layer-by-layer deposition (Figure S11). BY-2 bioinks were further introduced into shear-thinning supporting baths through a gel-in-gel process for suspension printing, a procedure similarly employed with a range of other inks (Figure 2F). Proliferation of cells within microgels was identified by the visible growth and via weight labeling, which showed a 6.25-fold increase in biomass after 7 days (Figure 2G).

**Figure 3:**
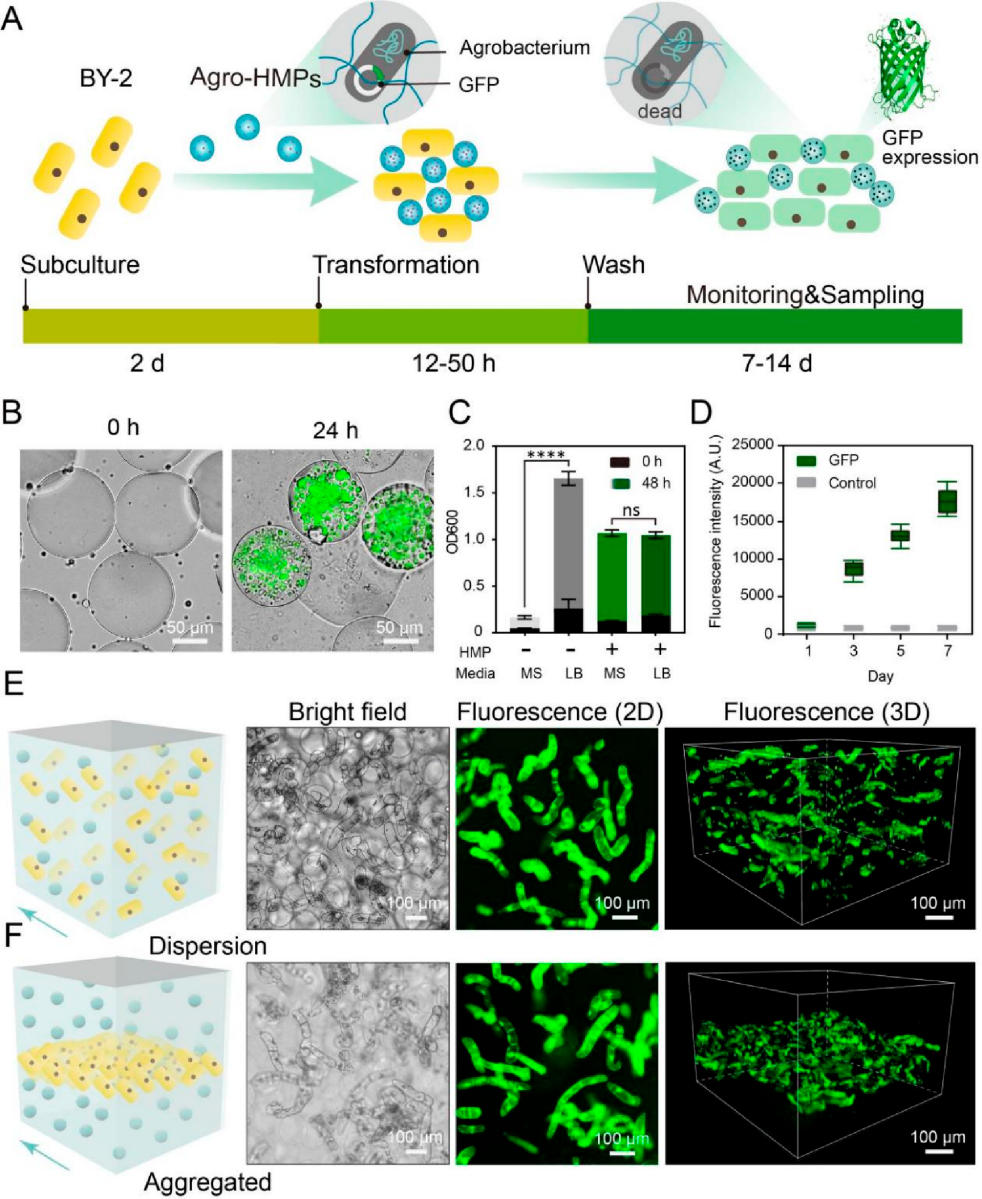
Generation of EPLM via HMP-facilitated *Agrobacterium* transformation. (A) Illustration of the workflow. BY-2 cells were subcultured and allowed to grow for 2 days before being mixed with Agro-HMPs and printed into scaffolds. After 12–50 h of inoculation, scaffolds containing BY-2 and live Agro-HMPs were washed in liquid media containing antibiotics to eliminate the *Agrobacterium* cells, giving rise to bacteria-free EPLM capable of proliferating and producing products-of-interest (GFP was used as an example). (B) Agro-HMPs 0 and 24 h after HMP formation. *Agrobacterium* were stained with live/dead assays to visualize growth after 24 h. Live and dead cells are shown as green and red, respectively. (C) OD600 of *Agrobacterium* cells grown in different bulk media with/without being embedded within HMPs after 24 h of growth. (D) GFP fluorescence intensity of BY-2 cells after transformation. (E) Bright and (F) fluorescence microscope images of suspension-printed ELPMs 14 days after transformation. Agro-HMPs carrying GFP were evenly distributed in the suspension buffer, and BY-2 cells were printed into (E) the whole area or in (F) a specific area. 2D fluorescence images showed similar patterns, whereas specific spatial distribution could be observed from 3D fluorescence images. Arrows indicate the direction of imaging.

Given the robust growth of BY-2 cells in granular hydrogel scaffolds, we explored the possibility of integrating engineered BY-2 cell lines to introduce novel functionalities and fabricate EPLMs. *Agrobacterium tumefaciens* has been frequently used to deliver genes-of-interest (GOIs) into a wide range of plant species including the tobacco BY-2 cell line.^24,25^ To develop an efficient foreign DNA delivery strategy into BY-2 cells within EPLMs, we capitalized on the success of HMPs to create injectable microporous scaffolds that have shown to significantly improve the transfection efficiency of infiltrated cells.^26^ To this end, *Agrobacterium* cultures were transformed with GOIs via electroporation, embedded within HMPs as Agro-HMPs, and then mixed with BY-2 cells and Gel-MA into bioinks (Figure 3A).

Embedded *Agrobacterium* cells were able to localize and proliferate within the HMPs after 24 h, as confirmed by live/dead staining assays (Figure 3B). Remarkably, growth rates of *Agrobacterium* cells embedded within LB-dissolved HMPs in either LB or MS liquid media were similar to those freely grown in the LB media and significantly higher than those freely grown in MS (Figure 3C). Agro-HMPs carrying genes encoding GFP were mixed with BY-2 cells, and green fluorescence could be observed from BY-2 cells 48 h after incubation (Figure S12). An extended infection period has been shown to adversely impact BY-2 growth (Figure S13).

Therefore, an incubation of 48 h was selected, after which MS media containing 25 μg/mL ampicillin was applied to wash the transformed scaffolds and eliminate *Agrobacterium* cells within Agro-HMPs (Figure S13), minimizing biofouling and preventing *Agrobacterium* leaching from the HMPs (Figure S14). GFP fluorescence intensity from EPLMs continuously increased for a week after transformation (Figure 3D).`

This HMP-mediated *Agrobacterium* transformation strategy has several advantages over traditional methods of simply mixing *Agrobacterium* and BY-2 cultures: (i) *Agrobacterium* cells were confined within HMPs so spatially controlled transformation of BY-2 cells could be achieved; (ii) embedded *Agrobacterium* cells were easy to kill after the completion of transformation to avoid contamination (Figure S15); (iii) compatibility to the 3D printing workflow; and (iv) transformed EPLMs were ready to use directly after washing. Spatially controlled printing and transformation of EPLMs are important steps toward the fabrication of multifunctional EPLMs with complex morphology. To this end, Agro-HMPs carrying genes encoding GFP were first loaded into a customized 3D cell culture chamber. Subsequently, BY-2 cells were dispensed in the entire chamber area (Figure 3E) or specifically printed in the central layer (Figure 3F), producing fluorescent patterns depending on the distribution of engineered BY-2 cells. Spatially targeted transformation of BY-2 cells was facilitated by the strategic deposition of Agro-HMPs within certain areas of the cell culture chamber (Figure S16), showing the system’s proficiency in crafting EPLMs with intricate spatial configurations.

**Figure 4:**
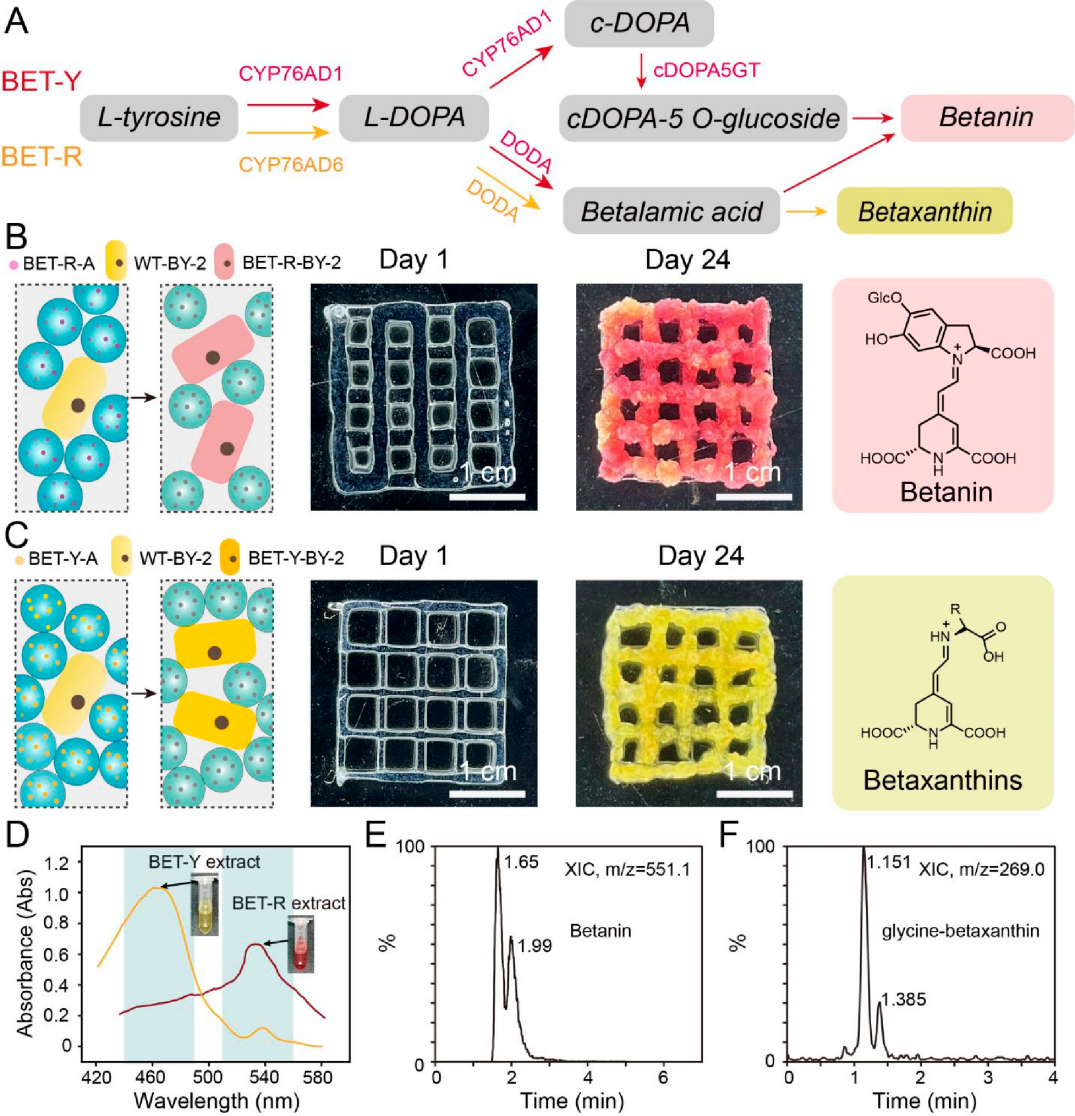
Construction of EPLMs capable of producing natural colorant betalains. (A) The biosynthetic pathway of betalains. l-Tyrosine (l-Tyr) is hydroxylated by cytochrome P450 CYP76AD1 or CYP76AD6 into l-DOPA. l-DOPA is converted into cyclo-DOPA (c-DOPA) by CYP76AD1 or into betalamic acid by DOPA 4, 5-dioxygenase (DODA). Spontaneous condensation of betalamic acid with amino acids or other amine groups generates yellow pigment betaxanthins. c-DOPA is glycosylated into cDOPA 5-*O*-glucoside by cyclo-DOPA-5-*O*-glucosyltransferase (cDOPA5GT) and undergoes spontaneous condensation with betalamic acid to form the red pigment betanin. Enzymes highlighted in red and yellow indicate those used in the BET-R and BET-Y constructs, respectively. (B) 3D printing of bioinks consisting of BET-R Agro-HMPs (BET-R-A) and wild-type BY-2 cells (WT-BY-2) into EPLMs. Transformed BY-2 cells (BET-R-BY-2) produced betanin, and the printed scaffold displayed red pigmentation after 24 days. (C) 3D printing of bioinks consisting of BET-Y Agro-HMPs (BET-Y-A) and WT-BY-2 into EPLMs. Transformed BY-2 cells (BET-Y-BY-2) produced yellow betaxanthins, and the printed scaffold displayed yellow pigmentation after 24 days. (D) Absorbance spectrum of extracts from BET-R and BET-Y BY-2 cells. (E) LC-MS analysis of extracts from BET-R scaffolds. Extracted ion chromatogram (XIC) showed the presence of the red pigment betanin (M + H = 555.1). (F) LC-MS analysis of extracts from BET-Y scaffolds. XIC showed the presence of the yellow pigment glycine-betaxanthin (M + H = 269.0).

The integration of our EPLMs, featured with customizable 3D shapes and spatially controlled transfection, with fast-growing synthetic biology tools creates lots of novel opportunities such as the in vitro studies of cell-to-cell communications in an artificial environment and the design of plant-based biofabrication hub for secondary metabolite production. Betalains are a class of red-violet or yellow pigments found in the Caryophyllales and possess high commercial values as natural colorants and dietary supplements.^27−29^ The biosynthetic pathway of betalains starts from a common substrate, l-tyrosine, and produces visible pigments in only 2 or 3 steps (Figure 4A). The simplicity of this pathway and valuable bioactivity of the products motivated us to use the betalain biosynthetic pathway as a model to explore the potential of EPLMs in synthetic biology applications.

Genes encoding betalain biosynthetic enzymes were cloned into three constructs, namely, BET-Y (Betalain-Yellow, containing *BvCYP76AD6* and *BvDODA*) for the production of yellow pigment betaxanthins, and BET-R-1 (Betalain-Red-1, containing *BvCYP76AD1* and *BvDODA*) and BET-R-2 (containing *MjcDOPA5GT*), which in combination produced the red pigment betanin (Figure 4A). BET-R-1 was not sufficient to drive strong red pigmentation on its own in our experiments, so mixture of *Agrobacterium* cultures carrying BET-R-1 and BET-R-2 were used in all following experiments for betanin production, named BET-R. Upon *Agrobacterium*-mediated infiltration, BET-R and BET-Y were shown to effectively induce the production of red and yellow pigmentations, corresponding to betanin and betaxanthin, in *N. benthamiana* leaves as previously reported (Figure S17).^30^ Agro-HMPs carrying BET-R and BET-Y were then made and extrusion printed together with BY-2 cells into grid-patterned structures. The generated EPLMs were able to grow over a period of 24 days without contamination and displayed corresponding red or bright yellow colors for those transformed with BET-R or BET-Y Agro-HMPs, respectively (Figures 4B,C and S18). BY-2 extracts from the BET-R and BET-Y EPLMs exhibited absorption peaks at 535 and 485 nm, corresponding to the reported absorption wavelengths for betanin and betaxanthin, which were also confirmed by LC-MS (Figure 4D).^31,32^

**Figure 5:**
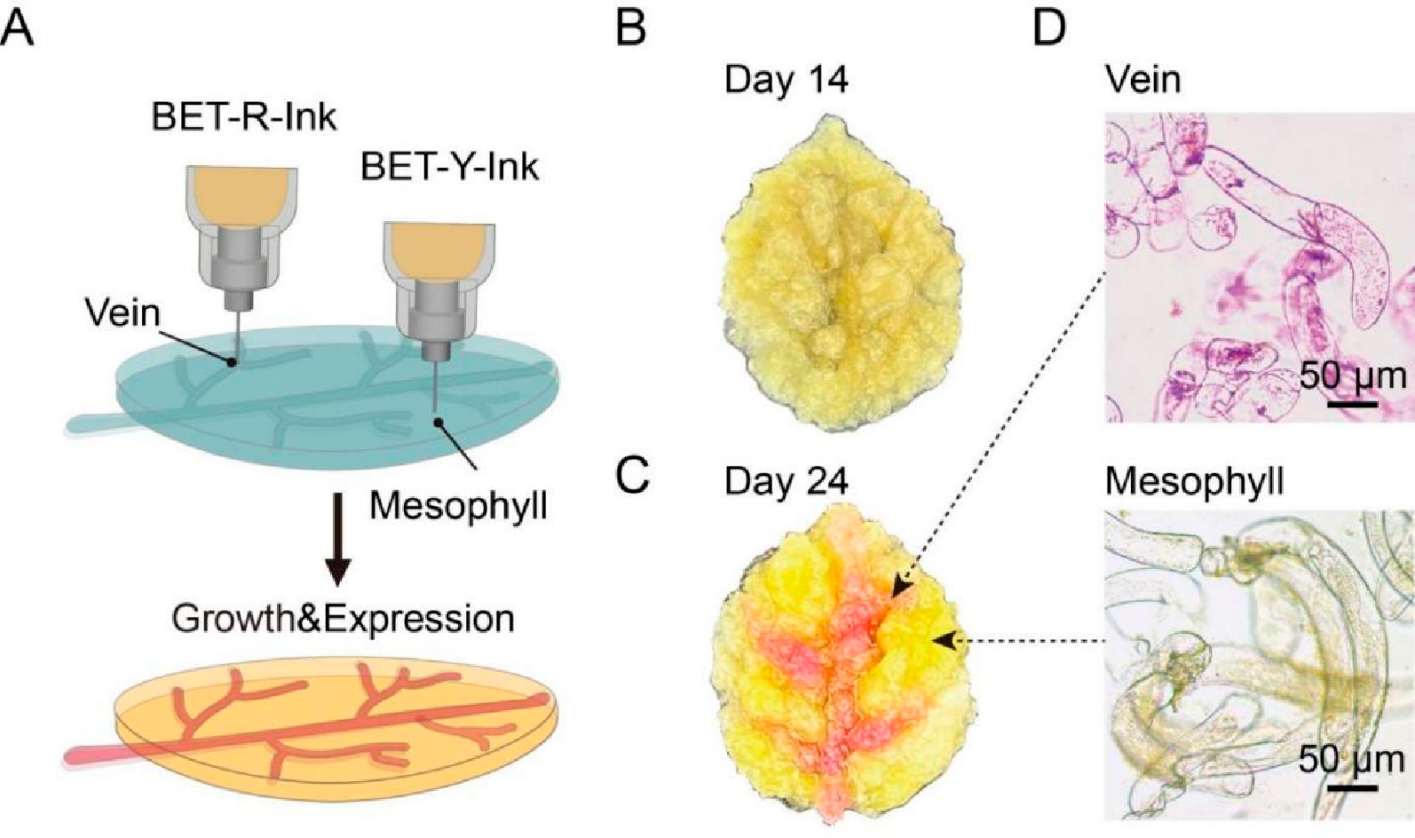
Construction of multifunctional EPLMs made of two distinct bioinks. (A) The fabrication of an artificial leaf-like scaffold was achieved by using two bioinks, namely, BET-R-Ink, containing Agro-HMPs bearing BET-R constructs, and BET-Y-Ink, containing Agro-HMPs bearing BET-Y constructs. BET-R-Ink and BET-Y-Ink were used to print the vein and mesophyll parts of the artificial leaf, respectively. (B) The artificial leaf-like scaffold expanded and became denser after a 14-day growth. Pigmentations were not yet visible at this stage. (C) After 24 days, chimeric patterns could be observed from the scaffold as a result of pigment production from different parts of the scaffold. (D) Microscopy images of BY-2 cells from the vein and mesophyll parts of the leaf-like scaffold.

The structural and functional configurations of the EPLMs were highly adaptable via 3D printing, allowing for the simultaneous application of various bioinks to create EPLMs with multiple functions. We demonstrated this capability by printing a leaf-like structure using two bioinks containing Agro-HMPs carrying BET-R and BET-Y as described above (Figure 5A). BET-R and BET-Y bioinks were used to print the vein and mesophyll parts of the leaf-like scaffold, respectively. BY-2 cells within the scaffold showed significant growth during 14 days of cultivation (Figure 5B), and chimeric patterns consisting of a red vein and bright yellow mesophyll parts of the artificial leaf-like scaffold could be observed after 24 days (Figure 5C). The accumulation of pigments within BY-2 cells resulted in the generation of cells displaying a red-violet or yellow color, respectively (Figure 5D). The transformable features of scaffolds allow cells within scaffolds to perform desirable transgenic expression. Therefore, specific functions and properties of artificial leaves could be customized by genetic reprogramming of different cells in the scaffold during the manufacture of artificial leaves. This is potentially important for advances in the fields of functional living materials and bioproduction of complex high-value chemicals.

## 3 Conclusion

In conclusion, we introduced a class of EPLMs that blend the realms of biology and engineering, pushing the boundaries of materials science. By utilizing tobacco BY-2 cells in combination with biocompatible HMPs, this study demonstrates the remarkable potential of 3D bioprinting technologies in creating biocompatible, structurally diverse, and functionally dynamic EPLMs. The superiority of using jammed HMPs is attributed to their enhanced printability, ability to create structurally and mechanically precise scaffolds, and potential for improved cell viability and function within the biofabricated materials. Further, the innovation of jammed HMP-mediated *Agrobacterium* transformation not only facilitates the growth of plant cells in 3D-printed scaffolds but also enables the introduction of foreign DNA, leading to the production of secondary metabolites and distinct pigmentation patterns as demonstrated in this study. These advancements signify a major step forward in the development of self-sustaining, responsive, and customizable living materials, with wide-ranging applications from sustainable construction to advanced biomanufacturing, opening up new possibilities for future technological and environmental solutions.

## 4 Materials and Methods

### Plant Materials and Growth Conditions

Wild-type *Nicotiana benthamiana* was grown in a controlled environment growth chamber (MGC-450HP-2 HengKexue Shanghai) with a 16 h photoperiod, 80% light intensity, 20 °C constant temperature, and 60% relative humidity. Plants were used for infiltration 5 to 6 weeks after germination. *N. tabacum* Bright-Yellow-2 (BY-2) cells were provided by Professor Tie’an Zhou at Hunan Agricultural University. BY-2 callus was cultured on Murashige and Skoog (MS-PhytoTech) media containing 3% sucrose, 0.2% KH_2_PO_3_, 0.002% glycine, 0.1% inositol, 0.1% vitamin B1, 0.02% (v/v) 2,4-dichlorophenoxyacetic acid (2,4-D), and 1% agar with a pH of 5.8. Suspended BY-2 cells were cultivated in the same medium without agar and agitated at 150 rpm. BY-2 callus and suspension cells were grown in a dark incubator at 26 °C.

### Synthesis of Gelatin Methacryloyl

Type A gelatin (Shanghai Aladdin Biochemical Technology) was dissolved in 50 °C deionized water to a concentration of 10 wt %. Methacrylic anhydride (0.6 g per gram of gelatin, Shanghai Macklin Biochemical) was added, and the reaction proceeded at 50 °C for 1 h with stirring. Termination involved adding 2 volumes of preheated 50 °C deionized water, followed by centrifugation at 3500*g* for 3 min. The supernatant was dialyzed (10 kDa molecular weight cutoff) in deionized water at 30 °C for 7 days. Postdialysis, Gel-MA was adjusted to pH 7.4 with 1 M NaOH. After dispensing, Gel-MA was snap frozen in liquid nitrogen and freeze-dried for 5–7 days.

### Synthesis of Pluronic F127

First, polyether F127 (90 mmol, 10 g) was added to a 250 mL flask and dried in a vacuum drying chamber at 80 °C for 4 h. After F127 cooled to room temperature, CHCl_3_ (0.99 mmol, 0.119 g) was added to the flask to dissolve F127 in an ice bath. C_6_H_15_N (7.87 mmol, 0.7968 g) was then added and stirred until the mixture was well mixed. To a constant-pressure buret containing 20 mL of CHCl_3_ (0.249 mmol, 0.0298 g) was added C_3_H_3_ClO (9.55 mmol, 0.11 g) in slow drops into the flask. Then, the reaction was carried out at room temperature for 48 h, and the precipitate was filtered off. After spin evaporation, the filtrate was added to an excess of C_4_H_10_O, and a white precipitate obtained by filtration. The precipitate was dissolved in a small amount of CHCl_3_ and then filtered after precipitation in C_4_H_10_O, and the resulting white solid was dried in a vacuum drying chamber.

### Synthesis of HB-PEGDA

Initially, PEGDA monomer (60 mmol, 575 g/mol) (Shanghai Aladdin Biochemical Technology) in 150 mL of butanone was transferred into a 250 mL flask. Initiators AIBN (3.4 mmol, 0.55 g) and DS (2.4 mmol, 0.71 g) were then introduced, followed by argon gas for 60 min. Stirring was continued at 70 °C for 6 h. Precipitation from ether/hexane (2:1, v/v) and subsequent drying under vacuum for 24 h finalized the purification process.

### Synthesis of SA-MA

A 1 g portion of sodium alginate (SA) (Aladdin) was dissolved in 50 mL of 45 °C deionized water, followed by addition of 7.14 mL of methacrylate anhydride (MA). A 5 mol/L NaOH solution was gradually added to keep the pH value of the polymer solution at 8 during the anhydride esterification process. After reaction at 0 °C for 24 h, the mixture was precipitated in ethanol and washed with ethanol to remove excess methacrylic acid. SA-MA was obtained by vacuum drying at 40 °C.

### Plasmid Construction

Gene sequences used in this study were *CYP76AD1* from *Beta vulgaris* (Genbank Accession MH836617),^33^*CYP76AD6* from *B. vulgaris* (Genbank Accession KM592962),^34^*DODA* from *B. vulgaris* (Genbank Accession MH836616),^33^ and *cDOPA5GT* from *Mirabilis jalapa* (Genbank Accession MH836618).^33^ BET-R and BET-Y were made using Golden Gate Cloning.^35^ The promoters (p) for each gene were as follows: *pCaMV35S:CYP76AD1*, *pCaMV35S:CYP76AD6*, *pCaMV35S:BvDODA*, *pAtUbi10:cDOPA5GT*, and *pCaMV35S:eGFP*.

### *A. tumefaciens* Transformation by Electroporation

*A. tumefaciens* strain GV3101 (Zoman Biotechnology) was transformed by electroporation. A 1 μL amount of plasmid DNA was added to 40 μL of electrocompetent GV3101 cells and transferred into a 2 mm electroporation cuvette (BioRad). Electroporation was carried out at 2.5 kV voltage, 25 μF capacitance, and 400 Ω resistance. A 600 μL amount of SOC media was added, and the culture incubated in a shaking incubator at 28 °C for 2 h. The culture was subsequently spread on LB plates containing appropriate antibiotic and grown at 28 °C for 2 days.

### Transient Transformation of *N. benthamiana* by Agroinfiltration

*N. benthamiana* plants were transformed by *Agrobacterium*-mediated infiltration using *A. tumefaciens* strain GV3101. *Agrobacterium* culture was grown overnight at 28 °C in LB media containing an appropriate antibiotic. Overnight culture was centrifuged at 2200*g* for 15 min, and the supernatant discarded. Pellets were resuspended in the infiltration buffer containing 10 mM MES (pH 5.6), 10 mM MgCl_2_, and 150 μM acetosyringone to a final OD600 of 0.5. *N. benthamiana* plants were filtrated by gently pushing the infiltration solution into the leaves with a syringe. Infiltrated plants were grown for 2–4 days before being harvested for analysis.

### Preparation of Gel-MA HMPs

Gel-MA microdroplets were generated in a flow-focusing microfluidic chip with a channel height of 100 μm and a junction dimension of 100 × 100 μm (Figure S2). A 10 wt % Gel-MA gel precursor solution (dissolved in MS liquid medium) and 0.5% blue light photoinitiator lithium phenyl-2,4,6-trimethylbenzoylphosphinate (LAP) were displaced into the device by syringe pumps (TYD01-02, Lead Fluid) via the middle channel. At the device junction, the hydrogel solution was sheared by the Novec 7500 fluorocarbon (3 M) containing 2% (v/v) Pico-surf surfactants (Sphere Fluidics). Flow rates of aqueous and oil phases were 8 and 40 μL/min, respectively, resulting in highly monodisperse droplets of ∼120 μm in diameter. Finally, 405 nm blue light was applied to initiate the cross-linking among microgels to generate HMPs.

### Preparation of HMP Encapsulating GV3101

To generate droplets of the water-in-oil system, two syringe pumps (TYD01-02, Baoding Lead Fluid Technology Co., Ltd., China) were injected into the microfluidic device. For the aqueous phase, 10 wt % Gel-MA gel precursor solution (dissolved in LB liquid medium) was selected, and 1 wt % *Agrobacterium* solution and 0.5% blue light photoinitiator LAP were added. The continuous oil phase was prepared by dissolving 2 wt % PicoSurf-1 in 3 M Novec 7500 perfluorinated oil. The flow rates of aqueous and oil phases were 8 and 40 μL/min, respectively.

### Extrusion Printing

Extrusion printing was conducted by using a commercial 3D bioprinter (EFL-BP-6601 Suzhou Yongquan Intelligent Equipment Co., Ltd., China). The bioink for printing was a mixture of 10 wt % Gel-MA (dissolved in MS) + BY-2 cells: HMPs = 1:2 (v/v), supplemented with 0.5 wt % LAP. The bioink was loaded into a 30 mL syringe, and temperatures of both the syringe and the printing platform were controlled using a pneumatic low-temperature platform, set at 0 and 5 °C, respectively. As the temperature inside the cylinder decreased, the ink exhibits ideal shear thinning and stress yield behavior due to the temperature sensitivity of Gel-MA and particle gel’s jamming state. Consequently, the samples uniformly extrude through a precision nozzle with an inner diameter of 0.77 mm under an atmospheric pressure of 0.6 psi. The printing speed is set at 800 mm/min, resulting in deposition dimensions on the slide (14.8 × 14.8 mm × 1.98 mm). Finally, by utilizing a blue light curing device (strength = 30 mW cm^–2^, 300–400 nm) for a duration of 300 s, a stable scaffold was obtained. All print paths were created by using CAD software (AutoCAD, Autodesk).

### Suspension Printing

Fluorescent dye molecules (rhodamine B-acrylate or fluorescein *o*-acrylate) were added into the original inks for better visualization effect. The suspended substrate consisted of 22 wt % Pluronic F127 dissolved in MS solution at 4 °C. The substrate was preheated to 30 °C before printing, which allowed it to transition into a gel state due to temperature sensitivity. The ink was then extruded at low temperatures through an 800 μm diameter nozzle and subsequently deposited onto the matrix. Blue light curing was performed as described above.

### HMP-Mediated *Agrobacterium* Transformation of BY-2 Cells

3D-printed scaffolds containing BY-2 cells and *Agrobacterium*-loaded HMPs were generated as described above, transferred to MS liquid media, and incubated for 48 h. The liquid culture was supplemented with 10 mM MES (pH 5.6), 10 mM MgCl_2_, and 150 μM acetosyringone to facilitate transformation of BY-2 cells. Scaffolds then were transferred to new MS liquid medium containing 25 μg/mL ampicillin after transformation to wash the scaffolds and kill *Agrobacterium* cells and then transferred to a new solid MS medium.

### Extraction of Betalain from Scaffolds

Scaffolds were snap frozen in liquid nitrogen for 20 s and thawed three times. A 1 mL sample of extraction solution consisting of 80% (v/v) ethanol and 1% (v/v) formic acid dissolved in deionized water was then added. The solution was sonicated for 30 min and centrifuged at 5000 rpm for 5 min, and the supernatant was collected into a new tube. Extracts were stored at 4 °C until use.

### Liquid Chromatography Mass Spectrometry (LC-MS) Measurement of Betalains

LC-MS spectrometry was performed on an Agilent 1290II-6460 equipped with a diode array detector (Agilent Technologies, Santa Clara, CA, USA), using an Agilent Eclipse Plus-C18 column (2.1 mm inner diameter × 100 mm, 1.8 μm). BET-R samples were run in a constant solvent consisting of solvent A (0.1% formic acid in water) and solvent B (100% methanol) in a 20:80 ratio, at a constant flow rate of 0.25 mL/min for 20 min. BET-Y samples were run using a gradient protocol as follows: 95% A (0–3 min), 95–75% A (3.0–10.0 min), 75–0% A (10–12 min), and 95% A (12–16 min). The injection volume of the sample was set at 5 μL. The detection wavelength was set at 535 nm for betanin and 480 nm for betaxanthins detection. Measurements were conducted in positive ion mode, and ion source conditions were as follows: ion source temperature 350 °C, nebulizer flow 10 L/min, nebulizer pressure 45 psi, fragmentation voltage 100 V, capillary voltage 4000 V.

### Confocal Microscopy

Confocal microscopy was performed on a Leica TCS STELLARIS 5 confocal microscope (Leica Microsystems Ltd., Breckland, UK) with a 10× air objective (HC PL APO CS2 10X 0.4 Dry). The excitation (λ_ex_) and emission (λ_em_) wavelengths of GFP were 488 and 561 nm.

### SEM Sample Preparation

ZEISS Sigma500 (Carl Zeiss GMBH) was used for the SEM analysis. For hydrogel sample preparation, hydrogel samples were frozen in liquid nitrogen and freeze-dried for 5 days. For cell sample preparation, cells were first collected and suspended in 1× PBS for 15 min, transferred to 3% glutaraldehyde (dissolved in 1× PBS) at 4 °C, and soaked overnight at 4 °C. After three washes, cells were soaked in 30%, 50%, 70%, 90%, and 100% ethanol for 15 min to replace glutaraldehyde and water and finally soaked in 90% and 100% isoamyl acetate for 20 min. Cell samples were then removed and freeze-dried for 5 days.

## Supporting Information Available

Testing of different bioink materials for optimal plant cell growth; microscopy image of generation of Gel-MA droplets by a microfluidic device; SEM images of hydrogel material; BY-2-loaded bioink for 3D printing; rheological characterization of jammed BY-2 bioink; optical and microscopy images of the growth of BY-2 within granular hydrogel scaffolds; optical images showcasing the preservation of structural integrity and rigidity throughout the growth of PLMs; SEM and light microscopy images showing the presence of HMPs throughout the cultivation of BY-2 cells; Gel-MA hydrogel weight enlargement versus time; optical and microscopy images of the growth of BY-2 cells at different locations of the scaffolds; different shapes of PLMs fabricated by 3D bioprinting; expression of GFP in BY-2 cells following *Agrobacterium*-mediated transformation; effect of *Agrobacterium* inoculation time on BY-2 cells growth; leakage and presence of *Agrobacterium* after inoculation; contamination of BY-2 cells following *Agrobacterium* incubation; schematic diagram and fluorescent image of suspension-printed EPLMs, 14 days post-transformation; transient expression of betalain biosynthetic pathway in *N. benthamiana* leaves following *Agrobacterium*-mediated infiltration; growth of transformed and wild-type PLMs (PDF)

## ABBREVIATIONS

EPLM: engineered plant living materials
PLMs: plant living materials
HMPs: hydrogel microparticles
BY-2: Bright-Yellow-2
Gel-MA: gelatin methacrylate
Agro-HMPs: *Agrobacterium*-loaded HMPs
GOI(s): gene(s)-of-interest
BET-Y: Betalain-Yellow construct, containing *BvCYP76AD6* and *BvDODA*
BET-R-1: Betalain-Red-1 construct, containing *BvCYP76AD1* and *BvDODA*
BET-R-2: Betalain-Red-2 construct, containing *MjcDOPA5GT*
l-Tyr: l-tyrosine
c-DOPA: cyclo-DOPA
DODA: DOPA 4,5-dioxygenase
cDOPA5GT: cyclo-DOPA-5-*O*-glucosyltransferase
BET-R-A: Agro-HMPs carrying BET-R constructs
BET-Y-A: Agro-HMPs carrying the BET-Y construct
WT-BY-2: wild-type BY-2 cells
