# Supplementary material for: Advancing Engineered Plant Living Materials through Tobacco BY-2 Cell Growth and Transfection within Tailored Granular Hydrogel Scaffolds
Source: ACS Cent Sci. 2024 May 1;10(5):1094–104. doi: 10.1021/acscentsci.4c00338 (PMC11117683; doi:10.1021/acscentsci.4c00338)
Supplement: Supplementary file 1 — oc4c00338_si_001.pdf [file oc4c00338_si_001.pdf]

Supporting Information

Advancing Engineered Plant Living Materials through Tobacco BY-2 Cell Growth and Transfection within Tailored Granular Hydrogel Scaffolds

Yujie Wang [a], *Zhengao Di* [b,c],* *Minglang Qin* [a], *Shenming Qu* [a], *Wenbo Zhong* [a], *Lingfeng Yuan* [a], *Jing Zhang* [a], *Julian M. Hibberd* [b], *Ziyi Yu* [a,*]

[a] State Key Laboratory of Materials-Oriented Chemical Engineering, College of Chemical Engineering, Nanjing Tech University, 30 Puzhu South Road, Nanjing 211816, P. R. China.

[b] Department of Plant Sciences, University of Cambridge, Downing Street, Cambridge CB2 3EA, UK.

[c] Earlham Institute, Norwich Research Park, Norwich, NR4 7UG, UK.

* E-mail addresses: ziyi.yu@njtech.edu.cn , zhengao.di@earlham.ac.uk (Corresponding authors)

Table of Contents

Figure S1:	Testing of different bioink materials for optimal plant cell growth.	1
Figure S2:	A microscopy image of generation of Gel-MA droplets by a microfluidic device.	2
Figure S3:	SEM images of hydrogel material.	3
Figure S4:	BY-2-loaded bioink for 3D printing.	4
Figure S5:	Rheological characterization of jammed BY-2 bioink.	5
Figure S6:	Optical and microscopy images of the growth of BY-2 within granular hydrogel scaffolds.	6
Figure S7:	Optical images showcasing the preservation of structural integrity and rigidity throughout the growth of PLMs.	7
Figure S8:	SEM and light microscopy images showing the presence of HMPs throughout the cultivation of BY-2 cells.	8
Figure S9:	Gel-MA hydrogel weight enlargement versus time.	9
Figure S10:	Optical and microscopy images of the growth of BY-2 cells at different locations of the scaffolds.	10
Figure S11:	Different shapes of PLMs fabricated by 3D bioprinting.	11
Figure S12:	Expression of GFP in BY-2 cells following *Agrobacterium*-mediated transformation.	12
Figure S13:	Effect of *Agrobacterium* inoculation time on BY-2 cells growth.	13
Figure S14:	Leakage and presence of *Agrobacterium* after inoculation.	14
FigureS15:	Contamination of BY-2 cells following *Agrobacterium* incubation.	15
FigureS16:	Schematic diagram and fluorescent image of suspension printed EPLMs, 14 days post-transformation.	16
FigureS17:	Transient expression of betalain biosynthetic pathway in *Nicotiana benthamiana* leaves following *Agrobacterium*-mediated infiltration.	17
FigureS18:	Growth of transformed and wild type PLMs.	18

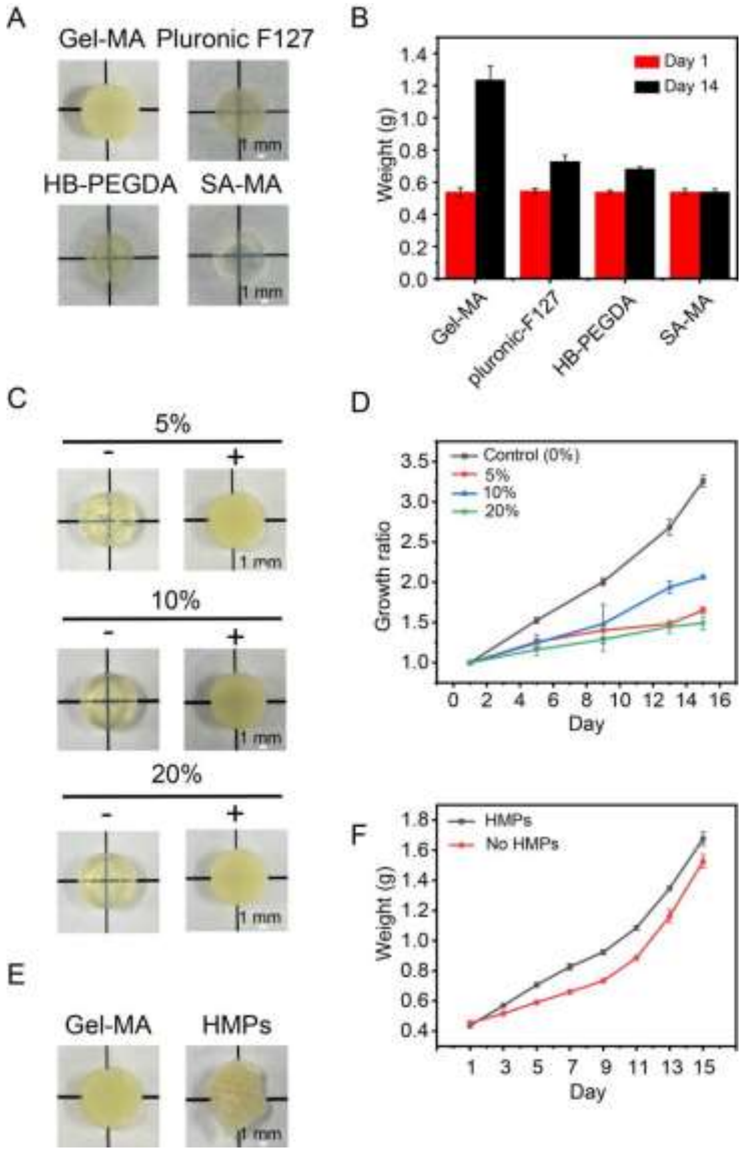

Figure S1. Testing of different bioink materials for optimal plant cell growth. A) Images of BY-2-loaded materials consisting of Gel-MA, Pluronic F127, HB-PEGDA, or SA-MA (10% wt) after 14 Days. Growth of BY-2 cells is indicated by the denser yellow color. B) Weights of BY-2-loaded materials at day 1 and day 14. C) Images of different concentrations of Gel-MA scaffolds with (+) or without (-) BY-2 cells 14 days after printing. D) Growth ratio (total weight on certain dates divided by weight on the first day) of BY-2 cells in the presence of different concentrations of Gel-MA. E) Images of scaffolds made by mixing BY-2 cells with Gel-MA or HMPs after14 days. F) Weight increases of BY-2 cells mixed in equal volumes of HMPs or Gel-MA (No HMPs).

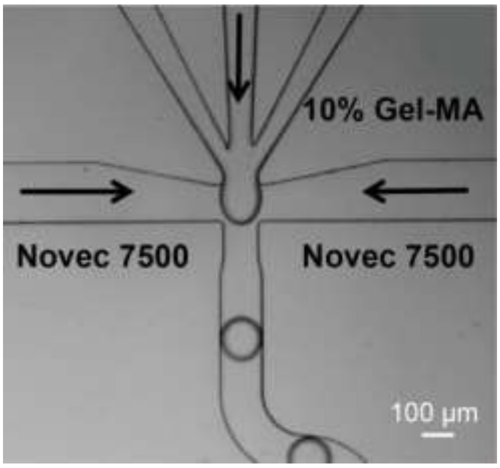

Figure S2. A microscopy image of generation of Gel-MA droplets by a microfluidic device.

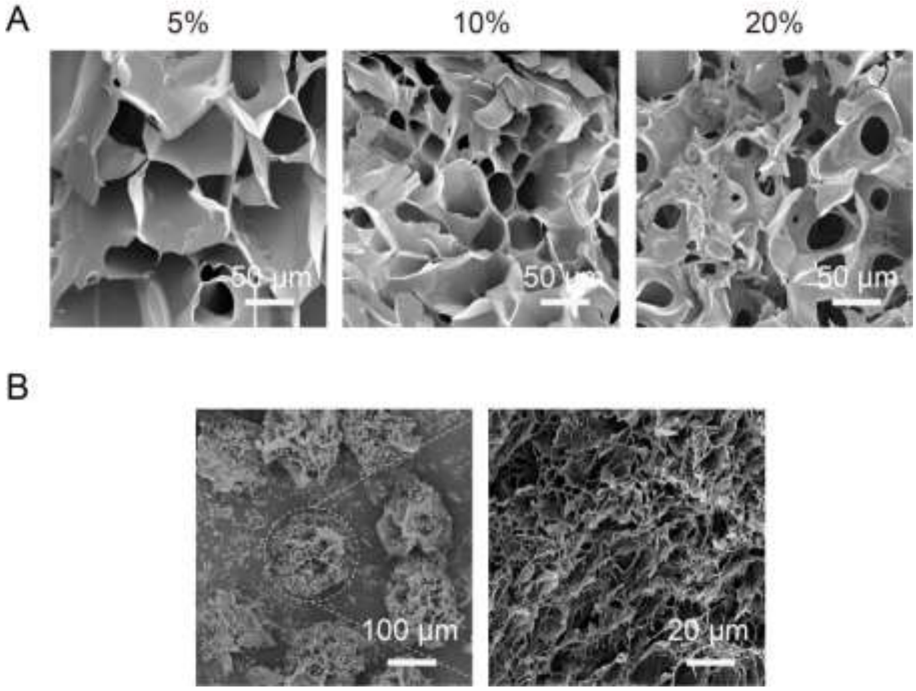

Figure S3. SEM images of hydrogel material. A) different Gel-MA concentrations. B) HMP structures.

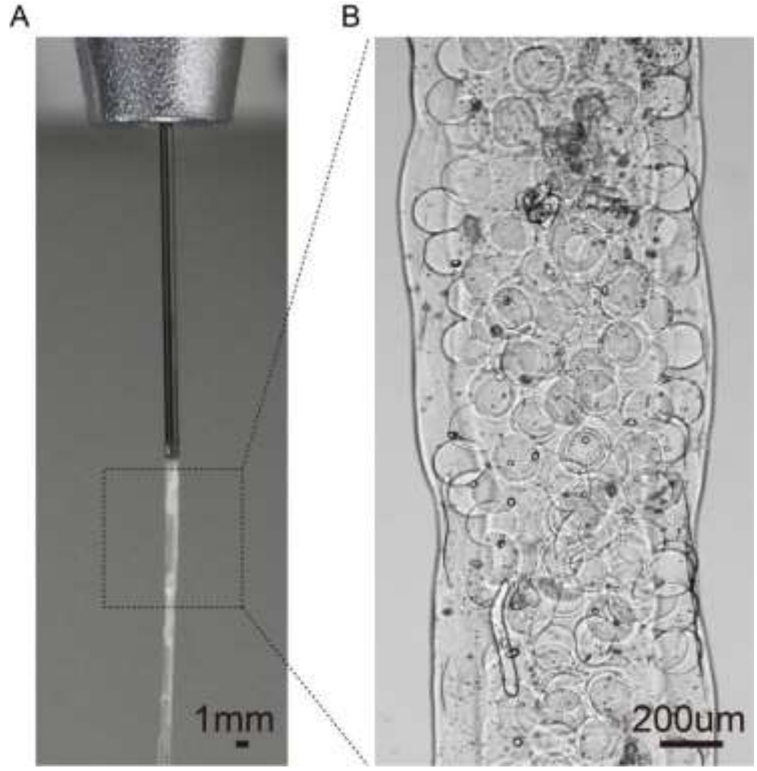

Figure S4. BY-2-loaded bioink for 3D printing. A) Images during jammed BY-2 bioink extrusion. B) Microscopic image of jammed BY-2 bioink after extrusion.

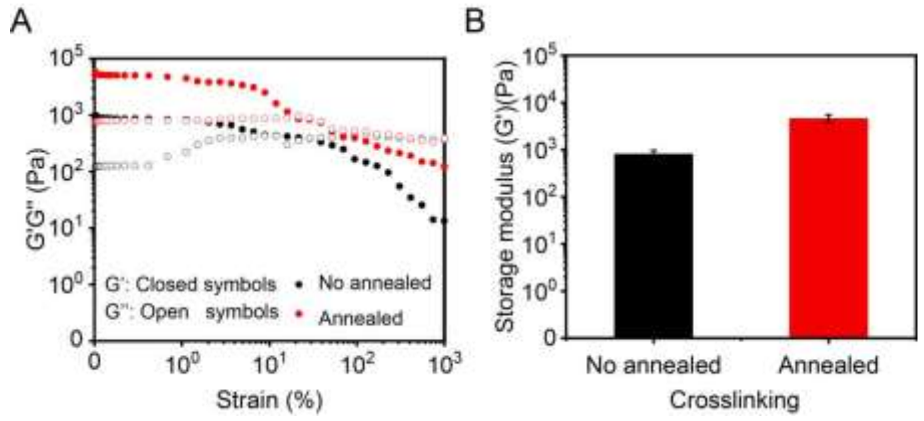

Figure S5. Rheological characterization of jammed BY-2 bioink. A) Storage moduli (G') and loss moduli (G") of jammed BY-2 bioink. B) Storage modulus (G') of the granular hydrogels. Black and red colors represent jammed BY-2 bioink with and without annealing, respectively.

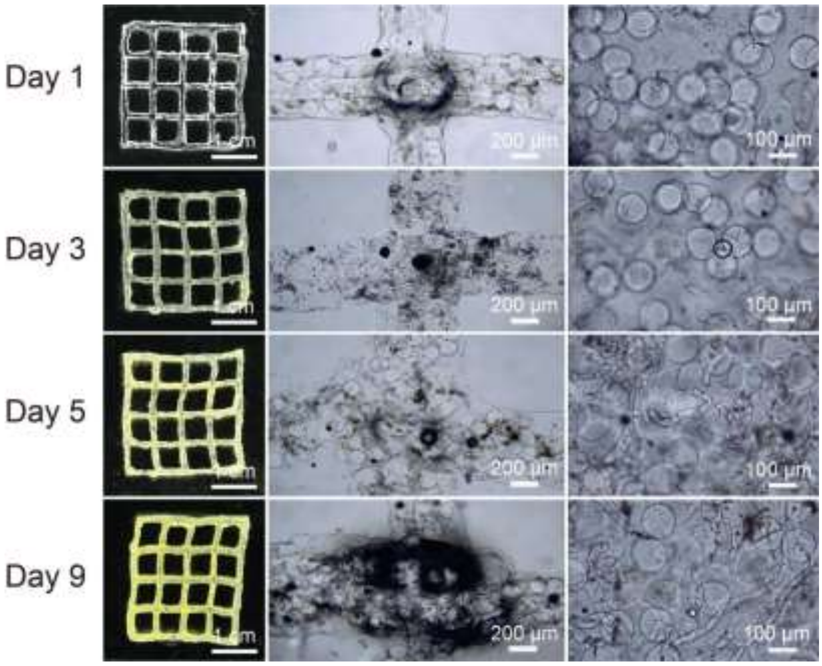

Figure S6. Optical and microscopy images of the growth of BY-2 within granular hydrogel scaffolds.

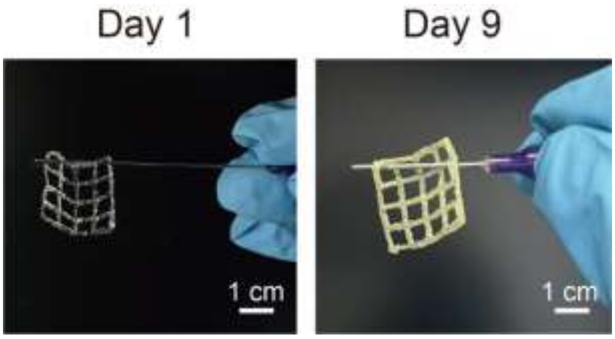

Figure S7. Optical images showcasing the preservation of structural integrity and rigidity throughout the growth of PLMs.

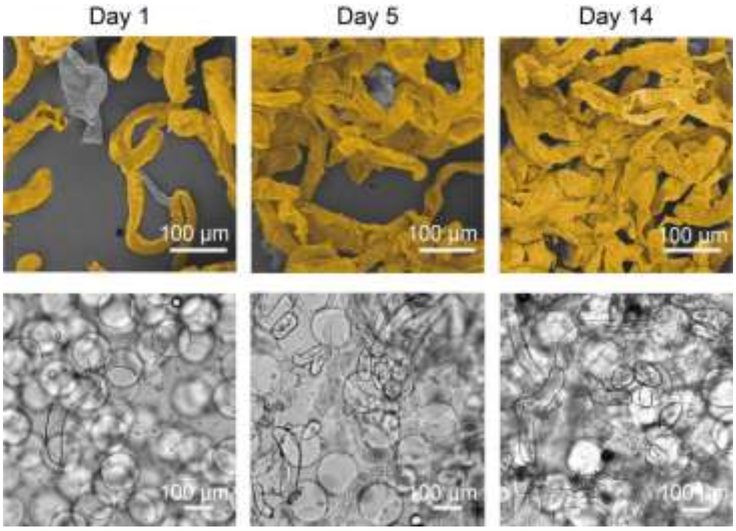

Figure S8. SEM and light microscopy images showing the presence of HMPs throughout the cultivation of BY-2 cells.

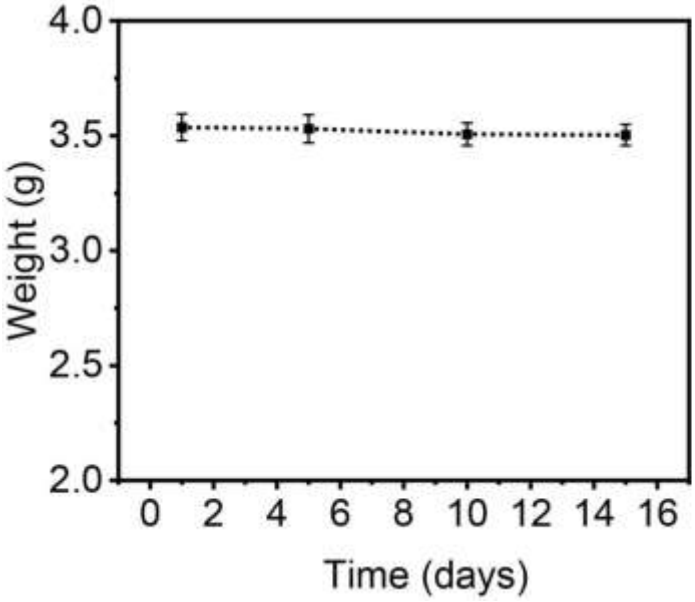

Figure S9. Gel-MA hydrogel weight enlargement versus time.

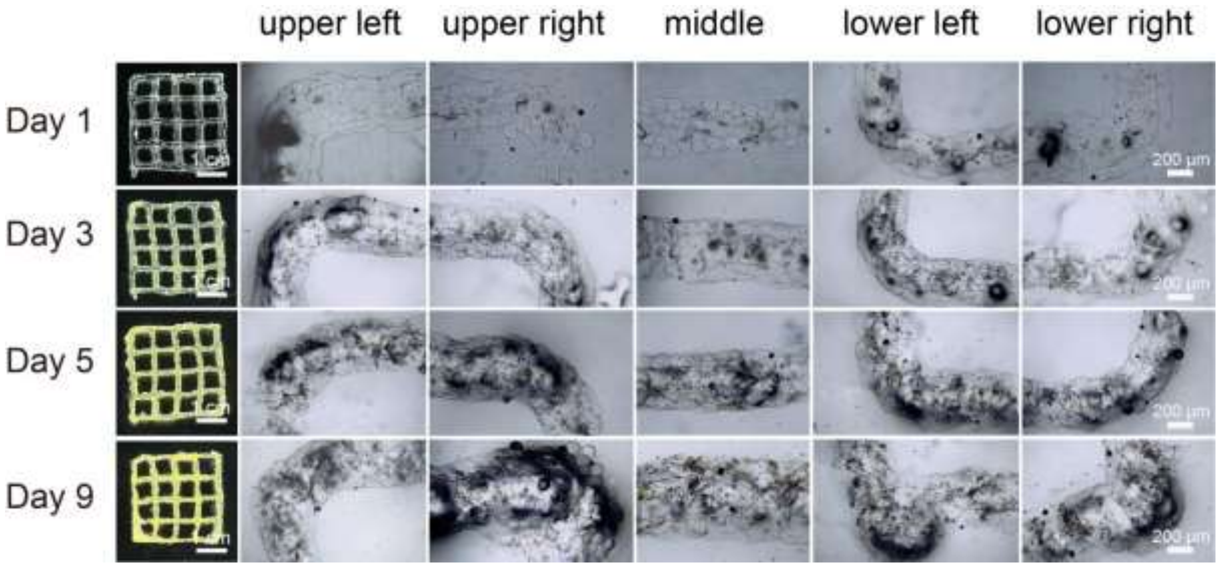

Figure S10. Optical and microscopy images of the growth of BY-2 cells at different locations of the scaffolds.

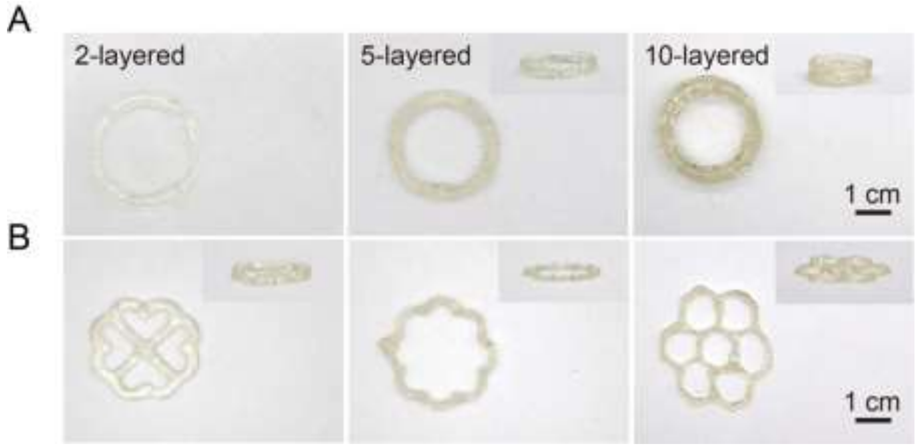

Figure S11. Different shapes of PLMs fabricated by 3D bioprinting. A) Side and top views of multi-layer circular PLMs prepared by layer-by-layer deposition. B) Side and top views of PLMs printed into various shapes.

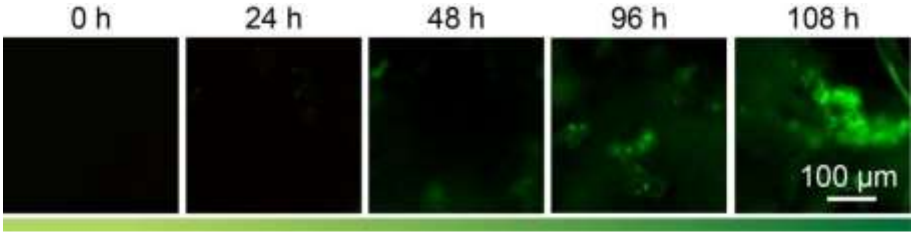

Figure S12. **Expression of GFP in BY-2 cells following *Agrobacterium*-mediated transformation.**

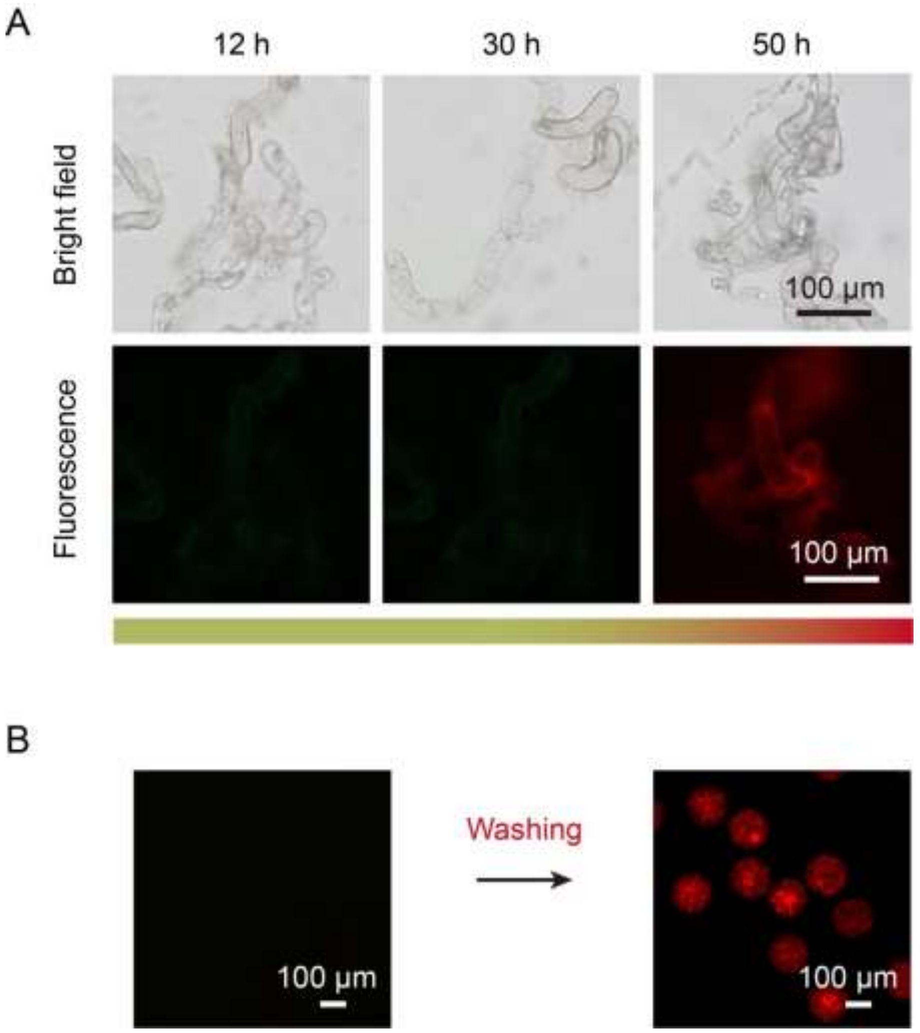

Figure S13. Effect of *Agrobacterium* inoculation time on BY-2 cell growth. A) Bright field (top row) and fluorescence (bottom row) images of BY-2 cells following different hours of *Agrobacterium* inoculation. Cells were stained ethidium homodimer-1 (EthD-1) and dead cells appeared red. B) Fluorescence images of EthD-1 stained *Agrobacterium* embedded in HMPs before and after washing in the MS media containing 25 µg/mL ampicillin.

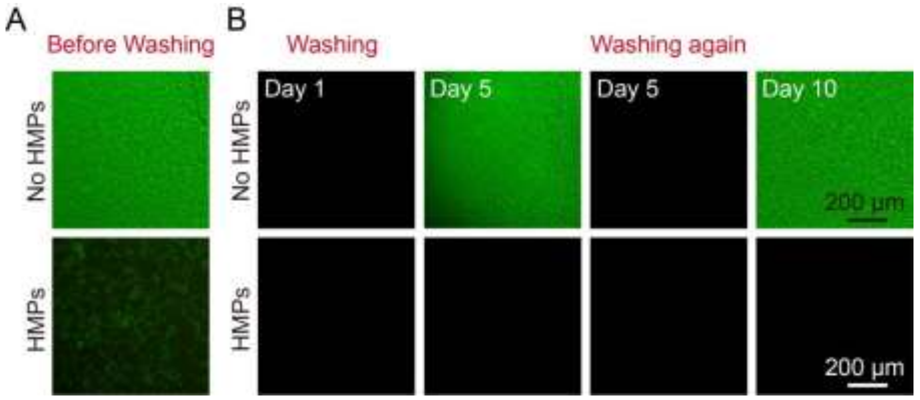

Figure S14. Leakage and presence of *Agrobacterium* after inoculation. No HMPs, *Agrobacterium* culture was added directly to BY-2 callus growing on solid media. HMPs, *Agrobacterium* were embedded in HMPs and mixed with BY-2 cells. A) After *Agrobacterium* inoculation, BY-2 samples were soaped in fresh MS liquid media for 30 min, and *Agrobacterium* leakage into the media visualized under fluorescence microscope. Cells were stained with calcein acetoxymethyl (calcein AM) and live cells appeared green. B) Transformed BY-2 cells were washed in MS media containing 25 µg/mL ampicillin and grown for 5 days. Then BY-2 cells were soaped in MS liquid media and released *Agrobacterium* observed under fluorescence microscope. C) The same washing-grow-soap procedure was repeated. *Agrobacterium* could still be observed from BY-2 callus transformed using traditional method (No HMPs) but not for HMP-mediated transformation method.

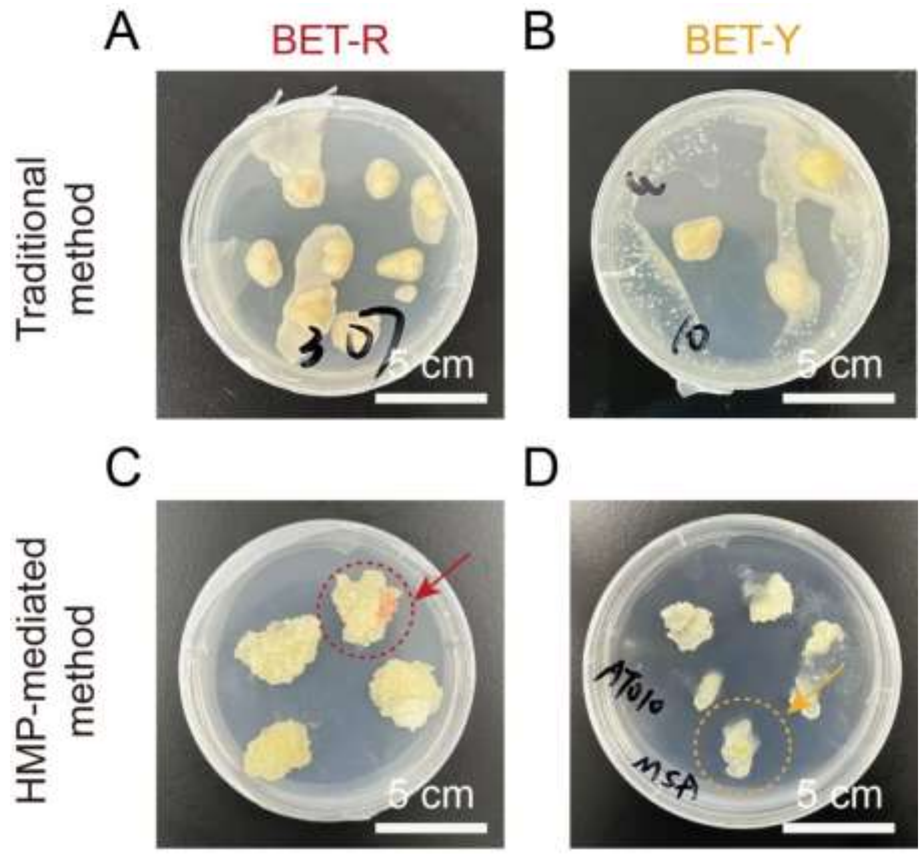

Figure S15. Contamination of BY-2 cells following *Agrobacterium* incubation. using traditional (A, B) or HMP-mediated (C, D) transformation methods. BET-R (A, C) and BET-Y (B, D) constructs were used and transformed cells showed red or yellow pigmentations, respectively (highlighted by circles and arrows).

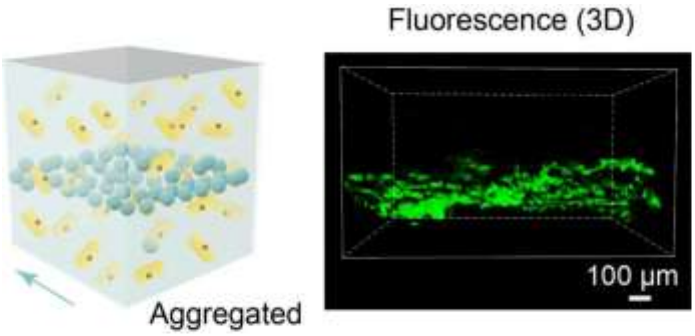

Figure S16. Schematic diagram and fluorescent image of suspension printed EPLMs, 14 days post-transformation. *Agrobacterium*-loaded HMPs (blue spheres in the left schematic illustration) carrying GFP were printed into the central layer of a BY-2 cell culture chamber (yellow squares in the left schematic illustration).

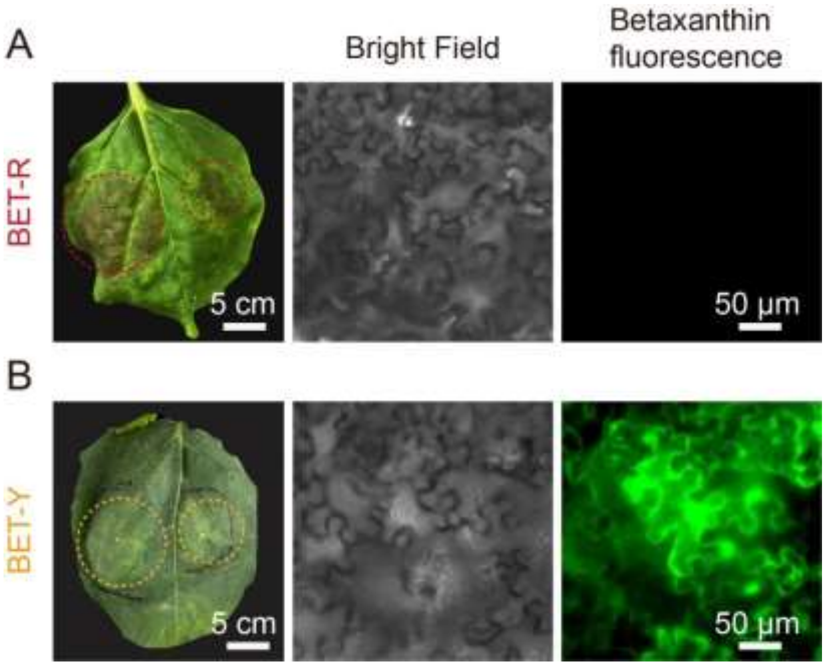

Figure S17. Transient expression of betalain biosynthetic pathway in *Nicotiana benthamiana* leaves following *Agrobacterium*-mediated infiltration. A-C) *N. benthamiana* infiltrated with mixture of BET-R-1 and BET-R-2 *Agrobacterium* cultures (BET-R). A) Infiltrated area turned red. No betaxanthin fluorescence was observed under confocal microscope. B) *N. benthamiana* infiltrated with *Agrobacterium* culture carrying BET-Y construct. Infiltrated area turned slightly yellow, which was partly masked by the green color of the leaf and not clearly visible by the eye. Fluorescence corresponding to betaxanthin could be observed under confocal microscope.

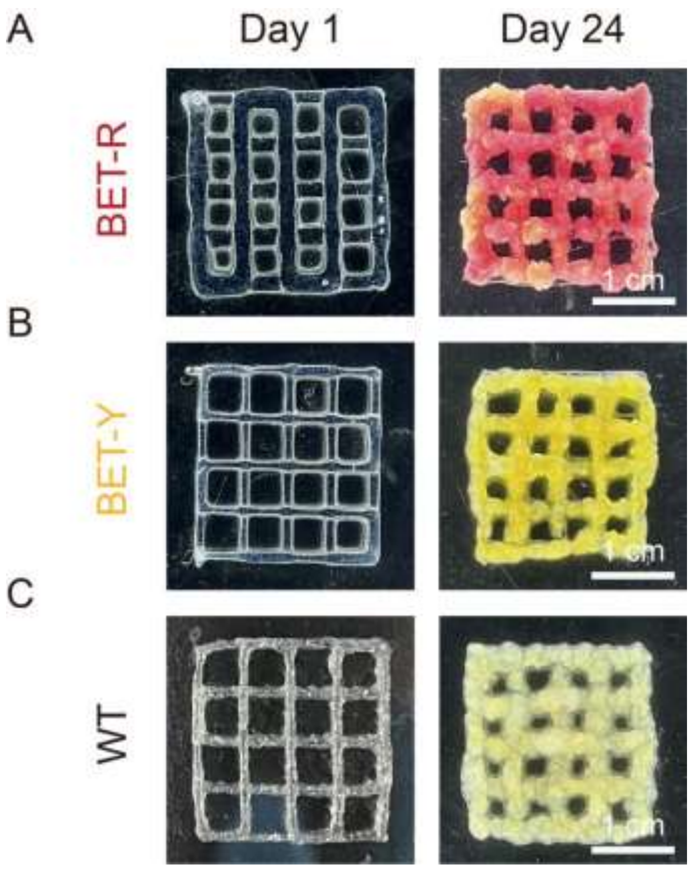

Figure S18. Growth of transformed and wild type PLMs. A) PLM printed from BET-R-Ink. The lattice scaffold grew denser and turned red after 24 days due to betanin production. B) PLM printed from BET-Y-Ink. The lattice scaffold grew denser and turned yellow after 24 days due to betaxanthin production. C) PLM printed from bioinks containing wild type *Agrobacterium*-loaded HMPs and BY-2 cells. The lattice scaffold grew denser and turned slightly pale yellow, which was the same color as observed from BY-2 cells growing in the liquid or solid MS media.
